# Employment of gene expression profiling to identify transcriptional regulators of hepatic stellate cells

**DOI:** 10.1186/1755-1536-5-S1-S12

**Published:** 2012-06-06

**Authors:** Hideaki Shimada, Lakshman E Rajagopalan

**Affiliations:** 1 Inflammation Research Unit, Pfizer Global Research and Development, Pfizer Inc, 700 Chesterfield Parkway West, Chesterfield, MO 63017, USA

## Abstract

Activated hepatic stellate cells (HSC) play a central role in scar formation that leads to liver fibrosis. The molecular mechanisms underlying this process are not fully understood. Microarray and bioinformatics analyses have proven to be useful in identifying transcription factors that regulate cellular processes such as cell differentiation. Using oligonucleotide microarrays, we performed transcriptional analyses of activated human HSC cultured on Matrigel-coated tissue culture dishes. Examination of microarray data following Matrigel-induced deactivation of HSC revealed a significant down-regulation of myocardin, an important transcriptional regulator in smooth and cardiac muscle development. Thus, gene expression profiling as well as functional assays of activated HSC have provided the first evidence of the involvement of myocardin in HSC activation.

## Introduction

Liver fibrosis is commonly observed after chronic liver injury and is believed to be a risk factor for cirrhosis and hepatocellular carcinomas (HCC). Fibrosis is a disease typified by the increased production and decreased degradation of extracellular matrix (ECM) surrounding hepatocytes [1,2]. Hepatic stellate cells (HSC) is play a key role in disease progression [3]. Following liver injury, quiescent vitamin A-containing HSC are activated and assume a myofibroblast-like phenotype characterized by proliferation, contractility and chemotaxis, accompanied by a progressive loss of stored vitamin A. Activated HSC are also trans-differentiate into α-smooth muscle actin (α-SMA)-positive, and produce excessive ECM, including type I collagen and fibronectin. Studies to date have demonstrated that several transcription factors, including KLF6 [4], c-Myb [5], Smad3 [6], MEF2 [7], FOXO1 [8], and PPARδ [9] are involved in HSC activation.

## Discussion

### Transcriptional analysis of HSCs

Omics technologies including genomics and proteomics are key tools in identifing the molecular mechanisms responsible for disease onset and progression. Gene expression profiling studies using DNA microarrays have been utilized to extensively characterize human fibrotic liver samples as well as livers from pre-clinical animal models of fibrosis. However, since quiescent stellate cells represent only 5-8% of total liver cells, it is difficult to examine accurately the gene expression changes in HSCs in whole liver. Culturing HSCs on plastic tissue culture dishes induces activation and this culture-induced activation has been employed as a model of HSC activation in liver fibrogenesis and profiled in multiple studies [10-15]. For example, Boer et al. [12] identified insulin-growth factor-binding proteins and gremlins as novel markers of liver fibrogenesis. Jiang et al. [11] identified the up-regulation of Wnt pathway signaling-related genes, Wnt5a and frizzled 2 in culture-induced activation and in development of liver fibrosis. These results clearly underline the usefulness of transcriptional analysis of activated HSC in categorizing molecular mechanisms responsible for liver fibrosis.

### Matrigel-induced HSC deactivation

**Figure 1:**
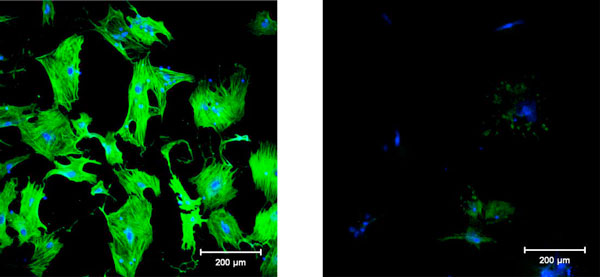
**Effect of Matrigel-induced deactivation on the morphology of activated rat activated HSCs**. Primary rat HSCs were plated either on plastic (left) or Matrigel-coated (right) tissue culture dishes and cultured for 3 days. Confocal microscopic images of HSC plated on plastic or Matrigel-coated dishes following staining with α-SMA antibody (green) and TOPRO-3 nuclear stain (blue). HSCs cultured on plastic dishes exhibit myofibroblast-like features, immunostaining for α-SMA, while cells plated on Matrigel-coated dishes stained weakly for α-SMA.

The ECM components regulate cellular process such as cell shape, motility, growth, differentiation and gene expression. BD Matrigel, a basement membrane matrix marketed by BD Biosciences is a gelatinous protein mixture secreted by Engelbroth-Hom-Swarm (EHS) mouse sarcoma cells and resembles the complex extracellular matrix environment. When cultured on plastic dishes, isolated quiescent HSC spontaneously transform to the activated state. However, culturing on Matrigel-coated dishes maintains the cells in a quiescent state [16]. Additionally, activated HSC can be deactivated on Matrigel, resulting in decreased α-SMA and collagen gene expression [17,18]. Figure 1. shows a comparison of rat primary HSC cultured either on plastic or Matrigel-coated dishes for 3 days, with Matrigel cultured stellate cells exhibiting a significantly reduced expression of α-SMA. Similarly, human HSC cell lines, LI90 and LX-2 cultured on Matrigel-coated dishes were deactivated, taking on around, compact appearance. Even though it is still unclear whether Matrigel can induce HSC reversion to a quiescent state, the cells cultured on Matrigel-coated dish were apparently deactivated.

Our previous expression profiling study using Affymetrix Human Genome U133 plus 2.0 Array demonstrated that 1044 genes including collagen genes were down-regulated in LI90 cell plated on Matrigel-coated dishes, while 2306 genes were up-regulated [19]. The over-represented Gene Ontology (GO) classification for down-regulated genes included categories related to 'muscle development' and 'cell growth'. The GO term 'muscle development' exhibited the lowest *P*-value of the biological processes and included the α-SMA gene. Several genes in the muscle development category exemplify the characteristics of myofibroblast-like cells and are expressed by smooth muscle cells. The down-regulation of muscle development-related genes was the primary biological process resulting in Matrigel-induced deactivation. To identify key transcription factors, we used Genomatix, a bioinformatics tool and BiblioSphere analysis to demonstrate that 41 transcription factors were significantly down-regulated in LI90 cells plated on Matrigel-coated dishes. Of these transcription factors, myocardin decreased 10-fold in LI90 cell. Knockdown or over-expression of myocardin significantly affected expression levels of muscle development-related genes [19], indicating that myocardin is a key transcriptional factor in Matrigel-induced HSC deactivation.

### Myocardin function in activated HSCs

Myocardin, a potent serum response factor (SRF) coactivator expressed in cardiac and smooth muscle cells, activates smooth muscle genes [20-22]. The SRF/myocardin complex has been reported to regulate the expression of muscle development-related genes such as α-SMA, CALD1, CNN1, and MYLK [23,24]. Myocardin can also function as a transcription coactivator by directly interacting with Smad3 to enhance SM22α promoter activity in a CArG box/SRF -independent manner [25,26], suggesting that myocardin may associate with Smad3 and thereby regulate collagen expression in HSCs. Knockdown of myocardin in human HSCs down-regulated some of the muscle development-related genes including α-SMA and collagens [19]. Herrmann et al. reported that TGF-β up-regulated SRF nuclear translocation and DNA-binding activity in activated HSC and simultaneously increased expression of the co-activator myocardin [27]. Targeted knockdown of SRF with RNAi decreased α-SMA expression in activated rat HSC [27]. In addition, our studies also demonstrated that myocardin gene expression was up-regulated during HSC activation of primary HSC and in fibrotic liver of dimethylnitrosamine (DMN)-induced fibrosis in a pre-clinical model [19]. These studies suggest a regulatory role for myocardin in both culture-induced HSC activation and Matrigel-induced HSC deactivation and in the pathogenesis of liver fibrosis.

While the role of myocardin in cardiac and smooth muscle development is well documented, recent studies have also implicated myocardin over-expression as being essential and sufficient for TGF-β mediated differentiation of human fibroblasts to myofibroblasts [28]. Liver fibroblasts participate in fibrogenesis by trans-differentiating into myofibroblast-like cells [29]. In our studies, myocardin over-expression induced the trans-differentiation of normal fibroblasts into myofibroblast-like cells with concomitant increase in α-SMA and COL1A1 gene expression [19]. We also found that myocardin increased SRF gene expression during trans-differentiation. SRF has a CArG box within the promoter region, which facilitates positive autoregulation. Accordingly, α-SMA gene expression, which is regulated by SRF, exhibited greater increases in expression than COL1A1 following myocardin over-expression. Our observations agree with previous findings [28] that TGF-β1-induced myocardin expression results in trans-differentiation of normal fibroblasts [28]. Thus, myocardin may play a key role in fibroblast trans-differentiation into myofibroblast-like cells during liver fibrosis. Further cellular localization studies will help to understand the function of myocardin in liver fibrosis. A quality antibody against myocardin is necessary and will enable sub-cellular localization studies to be performed.

## Conclusions

In summary, transcriptomics has become a key tool for the potential drug target and biomarker identification in the pharmaceutical industry. Omics technologies enable us to find key molecules responsible for disease. We have successfully identified myocardin as a transcription regulator involved in Matrigel-induced HSC deactivation. The myocardin pathway is therefore a promising new therapeutic target in the treatment of liver fibrosis. However, most transcription factors including myocardin are not readily druggable with small molecule inhibitors. To find more druggable targets, we need to map upstream to identify druggable upstream target is the RhoA associated kinase or ROCK [30]. In addition, emerging siRNA delivery technologies [31] may enable us to directly target myocardin mRNA.

## Competing interests

This work was funded by Pfizer Inc.
